# Supplementary material for: Improving Outcomes for Regional Families in the Early Years: Increasing Access to Child and Family Health Services for Regional Australia
Source: Int J Environ Res Public Health. 2024 Jun 4;21(6):728. doi: 10.3390/ijerph21060728 (PMC11203508; doi:10.3390/ijerph21060728)
Supplement: Supplementary file 1 [file ijerph-21-00728-s001.zip › ijerph-2970719-supplementary.pdf]

Table S1: Survey questions Family-centred care (8 items):

Did the staff member/s who worked with you:	
1. Take the time to understand the specific needs of you and your child	Never [ ]; seldom [ ]; sometimes [ ]; often [ ]; always [ ]
2. Respect that you are the expert on your child	Never [ ]; seldom [ ]; sometimes [ ]; often [ ]; always [ ]
3. Build your confidence as a parent	Never [ ]; seldom [ ]; sometimes [ ]; often [ ]; always [ ]
4. Ask you about how you are feeling as a parent	Never [ ]; seldom [ ]; sometimes [ ]; often [ ]; always [ ]
5. Address your concerns or questions about your child and give you specific information to address these concerns?	Never [ ]; seldom [ ]; sometimes [ ]; often [ ]; always [ ]
6. Understand you and your family and how you prefer to raise your child	Never [ ]; seldom [ ]; sometimes [ ]; often [ ]; always [ ]
7. Talk to you about resources that are available to support you (e.g. support groups, alternative health care)	Never [ ]; seldom [ ]; sometimes [ ]; often [ ]; always [ ]
8. Talk to you about issues in your community that may affect your child's health and development (e.g. the impact of drought, bushfires, floods or other issues impacting your community)	Never [ ]; seldom [ ]; sometimes [ ]; often [ ]; always [ ]

Table S2: Survey questions Goals, relationship and confidence questions (3 items)

1. Did the appointments with the nurses assist you in working towards goals to address your main reason for attending the service?	Yes [ ] No [ ] Unsure [ ]<br>Please explain…
2. Has your relationship with your child changed as a result of you attending the Tresillian service?	Yes [ ] No [ ] Unsure [ ]<br>Please explain…
3. Has your confidence as a parent changed as a result of you attending the Tresillian service?	Yes [ ] No [ ] Unsure [ ]<br>Please explain…

Table S3: Survey questions Service checklist (20 items)

Which of these services were you referred to, or encouraged to access, by the nurses at Tresillian? (*tick all that apply)*			
		(for those ticked, the following was asked)	
1. Gidget House	[ ]	**Have you attended this service or plan to attend this service?**	[ ] attended this service; [ ] plan to attend this service
2. Local playgroups	[ ]		[ ] attended this service; [ ] plan to attend this service
3. Child and Family Health Nurse service	[ ]		[ ] attended this service; [ ] plan to attend this service
4. Local family support services	[ ]		[ ] attended this service; [ ] plan to attend this service
5. Local support services – financial	[ ]		[ ] attended this service; [ ] plan to attend this service
6. Local support services – housing	[ ]		[ ] attended this service; [ ] plan to attend this service
7. Local support services – legal	[ ]		[ ] attended this service; [ ] plan to attend this service
8. Local support services – domestic violence	[ ]		[ ] attended this service; [ ] plan to attend this service
9. Local support services – drug and alcohol	[ ]		[ ] attended this service; [ ] plan to attend this service
10. Encouraged me to return to my GP (e.g. for further support with my physical or mental health needs or my child's health)	[ ]		[ ] attended this service; [ ] plan to attend this service
11. Tresillian residential services	[ ]		[ ] attended this service; [ ] plan to attend this service
12. Local health district Women's Health clinic	[ ]		[ ] attended this service; [ ] plan to attend this service
13. Lactation consultant	[ ]		[ ] attended this service; [ ] plan to attend this service

14. Local health district Mental health team	[ ]		[ ] attended this service; [ ] plan to attend this service
15. Local health district Perinatal mental health team	[ ]		[ ] attended this service; [ ] plan to attend this service
16. Physiotherapist	[ ]		[ ] attended this service; [ ] plan to attend this service
17. Occupational therapist	[ ]		[ ] attended this service; [ ] plan to attend this service
18. Speech therapist	[ ]		[ ] attended this service; [ ] plan to attend this service
19. Dietician	[ ]		[ ] attended this service; [ ] plan to attend this service
20. Social work	[ ]		[ ] attended this service; [ ] plan to attend this service
